# Esophageal fistula after definitive concurrent chemotherapy and intensity modulated radiotherapy for esophageal squamous cell carcinoma

**DOI:** 10.1371/journal.pone.0251811

**Published:** 2021-05-14

**Authors:** Tzu-Hui Pao, Ying-Yuan Chen, Wei-Lun Chang, Jeffrey Shu-Ming Chang, Nai-Jung Chiang, Chia-Ying Lin, Wu-Wei Lai, Yau-Lin Tseng, Yi-Ting Yen, Ta-Jung Chung, Forn-Chia Lin

**Affiliations:** 1 Department of Radiation Oncology, National Cheng Kung University Hospital, College of Medicine, National Cheng Kung University, Tainan, Taiwan; 2 Department of Oncology, National Cheng Kung University Hospital, College of Medicine, National Cheng Kung University, Tainan, Taiwan; 3 Department of Surgery, National Cheng Kung University Hospital, College of Medicine, National Cheng Kung University, Tainan, Taiwan; 4 Department of Internal Medicine, National Cheng Kung University Hospital, College of Medicine, National Cheng Kung University, Tainan, Taiwan; 5 National Institute of Cancer Research, National Health Research Institutes, Tainan, Taiwan; 6 Department of Diagnostic Radiology, National Cheng Kung University Hospital, College of Medicine, National Cheng Kung University, Tainan, Taiwan; Chang Gung Memorial Hospital and Chang Gung University, Taoyuan, Taiwan, TAIWAN

## Abstract

**Background:**

The literature regarding esophageal fistula after definitive concurrent chemotherapy and intensity modulated radiotherapy (IMRT) for esophageal squamous cell carcinoma (ESCC) remains lacking. We aimed to investigate the risk factors of esophageal fistula among ESCC patients undergoing definitive concurrent chemoradiotherapy (CCRT) via IMRT technique.

**Methods:**

A total of 129 consecutive ESCC patients receiving definitive CCRT with IMRT between 2008 and 2018 were reviewed. The cumulative incidence of esophageal fistula and survival of patients were estimated by the Kaplan–Meier method and compared between groups by the log-rank test. The risk factors of esophageal fistula were determined with multivariate Cox proportional hazards regression analysis.

**Results:**

Median follow-up was 14.9 months (IQR, 7.0–28.8). Esophageal perforation was identified in 20 (15.5%) patients, resulting in esophago-pleural fistula in nine, esophago-tracheal fistula in seven, broncho-esophageal fistula in two, and aorto-esophageal fistula in two patients. The median interval from IMRT to the occurrence of esophageal fistula was 4.4 months (IQR, 3.3–10.1). Patients with esophageal fistula had an inferior median overall survival (10.0 vs. 17.2 months, p = 0.0096). T4 (HR, 3.776; 95% CI, 1.383–10.308; p = 0.010) and esophageal stenosis (HR, 2.601; 95% CI, 1.053–6.428; p = 0.038) at baseline were the independent risk factors for esophageal fistula. The cumulative incidence of esophageal fistula was higher in patients with T4 (p = 0.018) and pre-treatment esophageal stenosis (p = 0.045). There was a trend toward better survival after esophageal fistula among patients receiving repair or stenting for the fistula than those only undergoing conservative treatments (median survival, 5.9 vs. 0.9 months, p = 0.058).

**Conclusions:**

T4 and esophageal stenosis at baseline independently increased the risk of esophageal fistula in ESCC treated by definitive CCRT with IMRT. There existed a trend toward improved survival after the fistula among patients receiving repair or stenting for esophageal perforation.

## Introduction

Esophageal cancer ranks seventh most common malignancy and sixth leading cause of cancer-related death globally [1]. Definitive concurrent chemoradiotherapy (CCRT) is one of the treatment options for locally advanced esophageal cancer [2, 3]. Esophageal fistula could occur as a consequence of tumor invasion or therapy-related tissue damage [4].

The development of esophageal fistula was found in 5.3–24.1% of patients undergoing chemoradiotherapy for esophageal cancer. The previously published researches included cases undergoing concurrent or sequential chemoradiotherapy with miscellaneous radiotherapy techniques [5–10]. Accordingly, esophageal fistula remains to be elucidated specifically in esophageal cancer after definitive concurrent chemotherapy and intensity modulated radiotherapy (IMRT).

In the present study, we analyzed a single-institution cohort of esophageal squamous cell carcinoma (ESCC) patients receiving definitive CCRT via IMRT technique. The cumulative incidence and risk factors of esophageal fistula were investigated. Moreover, we reported the clinical course of esophageal fistula which was less depicted in the literature.

## Methods

### Patients and study design

This study was approved by the Institutional Review Board of National Cheng Kung University Hospital (reference number, A-ER-107-349). All data were not anonymized before we accessed them. The informed consent was waived because of the retrospective nature of the study. Patients with ESCC treated by definitive CCRT at our institution between 2008 and 2018 were reviewed. Patients were recruited on the basis of criteria as follows: newly pathologically confirmed ESCC without distant metastasis, no prior thoracic radiotherapy, definitive CCRT via IMRT, and conventional radiotherapy fractionation with dose ≥ 50 Gy. The pre-treatment evaluation of esophageal cancer included esophagogastroduodenoscopy, endoscopic ultrasonography, computed tomography (CT) of the chest and abdomen, and bone scan. Positron emission tomography-computed tomography was performed in cases with indeterminate results of CT or bone scan. The clinical stage was classified according to the seventh edition of the American Joint Committee on Cancer staging system. Esophageal stenosis was defined as a narrowing of the esophagus which was caused by the tumor growth and could not be passed through by the endoscopy.

### Definitive concurrent chemoradiotherapy

All patients received definitive CCRT with IMRT technique as previously described [11–13]. Briefly, the clinical target volume (CTV) 1 included gross tumor volume (GTV) of the primary (GTVp) with a 5-cm craniocaudal and 1-cm radial margin along the esophagus, and GTV of lymph nodes (GTVn) with a 1-cm margin. The CTV 2 included GTVp with a 2-cm craniocaudal and 1-cm radial margin along the esophagus, and GTVn with a 1-cm margin. The planning target volume was generated by expanding 1 cm around the GTV and CTV in all directions. CTV 1 and 2 were sequentially treated to 36 and 50–50.4 Gy, respectively. Thereafter, GTV was boosted up to 66–66.6 Gy if dose constraints of the organs at risk could be met. Furthermore, chemotherapy, nutrition and supportive care were given during radiation treatment as previously described [12, 13].

### Evaluation of esophageal fistula

Follow-up evaluations included clinical examinations, esophagogastroduodenoscopy, and CT scan of the chest and abdomen at 1 month after CCRT and then every 3–6 months. In addition, bronchoscopy, esophagogram with water-soluble or barium contrast, CT angiogram, and other examinations were arranged as clinically indicated. To identify the occurrence and elucidate the clinical course of esophageal perforation, clinical symptoms and signs, esophagogastroduodenoscopy, CT images, bronchoscopy, esophagogram, managements for esophageal fistula, and outcomes were reviewed.

### Statistical analysis

The data cutoff date was July 17, 2020. Overall survival (OS) was calculated from the start of IMRT to the date of death or last follow-up. The time to esophageal fistula was defined as the interval from the beginning of IMRT to the occurrence of the event. Survival of patients and cumulative incidence of esophageal fistula were estimated by the Kaplan-Meier method and compared between groups by the log-rank test. The factors associated with esophageal fistula were checked with univariate analysis. The independent risk factors of esophageal fistula were examined by multivariate Cox proportional hazards regression analyses in which the variables with a trend in univariate analysis were taken into consideration. A p-value < 0.05 was considered statistically significant. Statistical analyses were performed with SPSS version 22.0 software and R version 3.5.1 for Windows.

## Results

### Characteristics of the included patients

Of the 204 patients reviewed, 129 patients matched the recruitment criteria while 78 the remaining patients were excluded from the analysis with reasons as follows: stage IV (n = 21), radiation dose < 50 Gy (n = 20), use of three-dimensional conformal radiation therapy (3DCRT) technique (n = 20), salvage esophagectomy for persistent tumor (n = 9), and histology other than squamous cell carcinoma (n = 5). Table 1 summarized demographic and clinical characteristics of the included five (3.9%) female and 124 (96.1%) male patients. One hundred and nineteen (92.2%) patients had stage III ESCC. The median radiation dose was 61.2 Gy (IQR, 54.0–66.6). Fluoropyrimidine-based chemotherapy regimens were used in 121 (93.8%) patients. Other regimens were utilized at the discretion of physicians (S1 Table).

**Table 1:** Demographic and clinical characteristics of patients at baseline.

Characteristic	No. of patients (%)
Age (years)	
Median (Range)	57 (34–81)
≤ 57 : > 57	69 (53.5) : 60 (46.5)
Gender	
Male : Female	124 (96.1) : 5 (3.9)
Body mass index (kg/m^2^)	
Median (Range)	21.1 (15.5–30.0)
≤ 21.1 : > 21.1	72 (55.8) : 57 (44.2)
Body surface area (m^2^)	
Median (Range)	1.61 (1.28–2.10)
≤ 1.61 : > 1.61	65 (50.4) : 64 (49.6)
Eastern Cooperative Oncology Group performance status	
0 : 1 : 2 : 3	12 (9.3) : 100 (77.5) : 16 (12.4) : 1 (0.8)
Stage	
I : II : III	2 (1.6) : 8 (6.2) : 119 (92.2)
T stage	
1 : 2 : 3 : 4	6 (4.7) : 12 (9.3) : 86 (66.7) : 25 (19.4)
N stage	
0 : 1: 2 : 3	5 (3.9) : 15 (11.6) : 40 (31.0) : 69 (53.5)
Tumor location	
U^a^ : M^b^ : L^c^	53 (41.1) : 37 (28.7) : 14 (10.9)
U + M (from U to M)	11 (8.5)
M + L (from M to L)	14 (10.9)
Esophageal stenosis	
Yes : No	47 (36.4) : 82 (63.6)
Total circumferential tumor	
Yes : No	12 (9.3) : 117 (90.7)
Tumor length (cm)	
Median (Range)	5.0 (1.1–15.0)
≤ 5.0 : > 5.0	73 (56.6) : 56 (43.4)
Smoking	
Yes : No	113 (87.6) : 16 (12.4)
Alcohol	
Yes : No	117 (90.7) : 12 (9.3)
Hypertension	
Yes : No	22 (17.1) : 107 (82.9)
Diabetes	
Yes : No	17 (13.2) : 112 (86.8)
Chemotherapy regimen	
Fluoropyrimidine-based	121 (93.8)
Taxane-based	6 (4.7)
Others	2 (1.6)
Radiation dose (Gy)	
Median (IQR)	61.2 (54.0–66.6)
≤ 61.2 : > 61.2	73 (56.6) : 56 (43.4)

^a^U: upper thoracic and cervical esophagus.

^b^M: middle thoracic esophagus.

^c^L: lower thoracic esophagus.

### Inferior overall survival in patients with esophageal fistula

**Fig 1:**
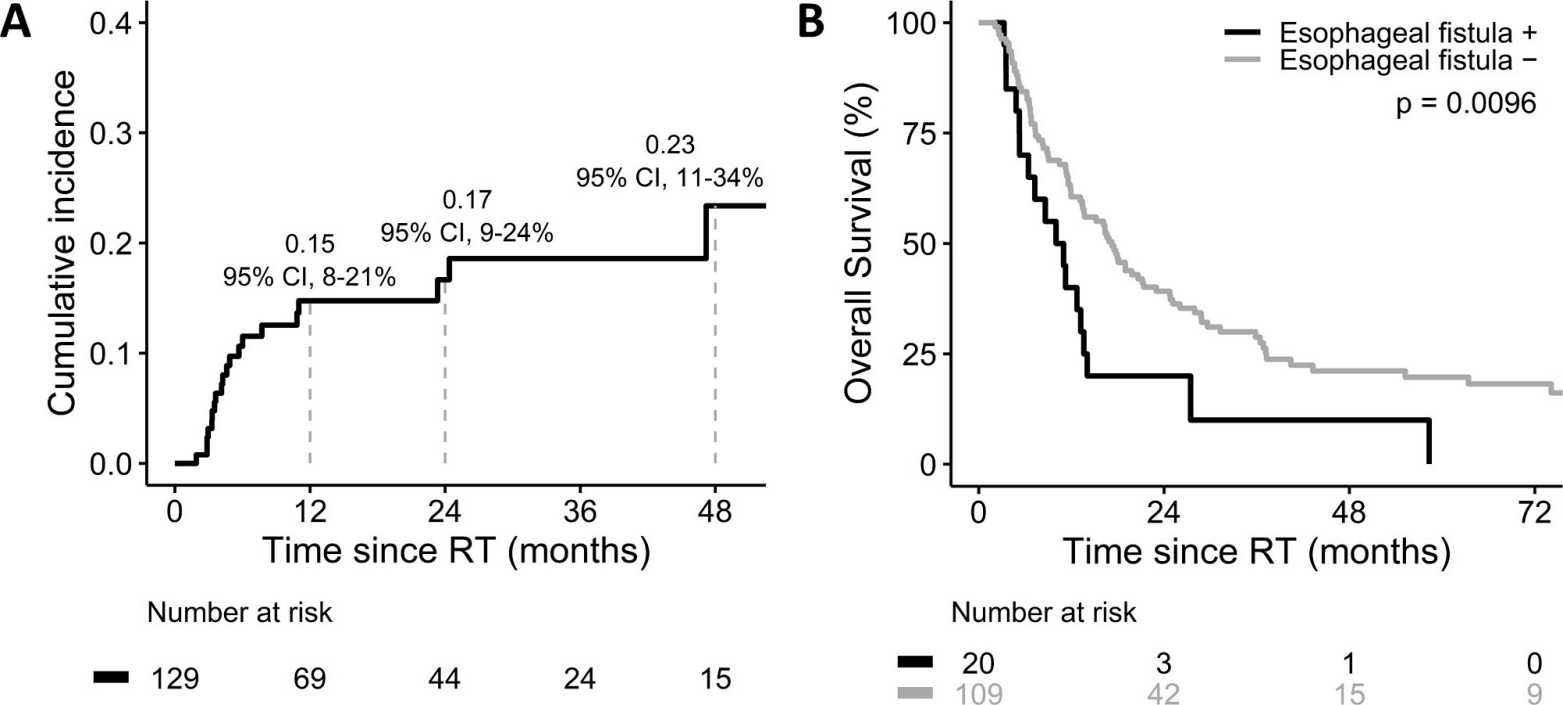
**(A) Cumulative incidence of esophageal fistula. (B) Overall survival by esophageal fistula**.

The median follow-up and OS were 14.9 (IQR, 7.0–28.8) and 15.2 months (IQR, 7.3–36.9), respectively. Esophageal perforation was identified in 20 (15.5%) patients, resulting in esophago-pleural fistula in nine, esophago-tracheal fistula in seven, broncho-esophageal fistula in two, and aorto-esophageal fistula in two patients. The median interval from IMRT to the occurrence of esophageal fistula was 4.4 months (IQR, 3.3–10.1). The cumulative incidence of esophageal fistula at one, two, and four years was 0.15 (95% CI, 0.08–0.21), 0.17 (95% CI, 0.09–0.24), and 0.23 (95% CI, 0.11–0.34), respectively (Fig 1A). Moreover, patients who experienced esophageal fistula had an inferior median OS (10.0 vs. 17.2 months, p = 0.0096; Fig 1B).

### Clinical characteristics associated with esophageal fistula

**Fig 2:**
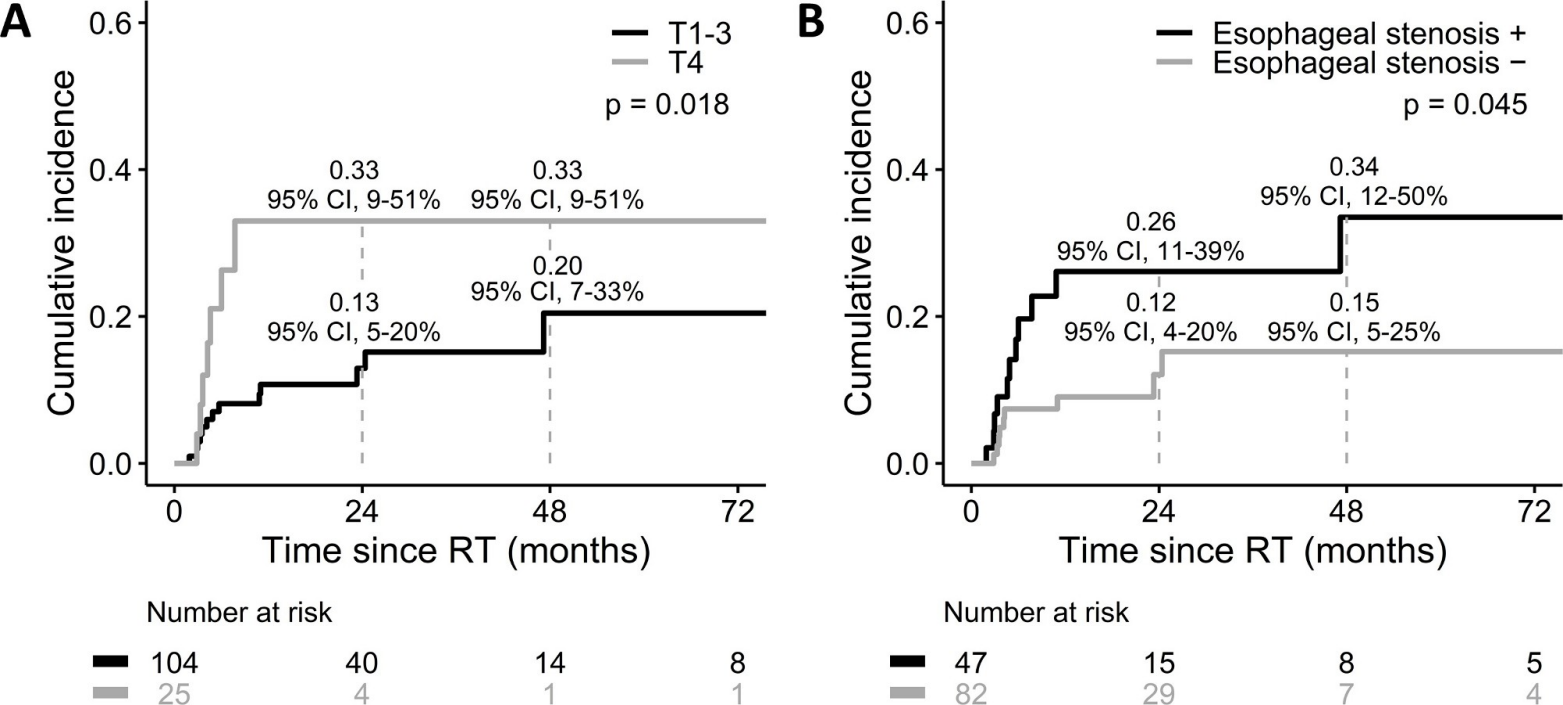
**Cumulative incidence of esophageal fistula by (A) T stage and (B) esophageal stenosis**.

We next investigated the factors associated with esophageal fistula through univariate analyses (Table 2). Five variables were further included in the multivariate Cox proportional hazards regression analysis. T4 (HR, 3.776; 95% CI, 1.383–10.308; p = 0.010) and esophageal stenosis (HR, 2.601; 95% CI, 1.053–6.428; p = 0.038) at baseline were the independent risk factors for esophageal fistula (Table 3). T4 correlated with more esophageal fistula (p = 0.018; Fig 2A). The cumulative incidence of esophageal fistula at two and four years was 0.33 (95% CI, 0.09–0.51) in patients with T4 whereas it was 0.13 (95% CI, 0.05–0.2) and 0.2 (95% CI, 0.07–0.33), respectively, among cases with T1-3. Moreover, esophageal fistula more frequently occurred in patients with pre-treatment esophageal stenosis (p = 0.045; Fig 2B). The cumulative incidence of esophageal fistula was 0.26 (95% CI, 0.11–0.39) at two years and 0.34 (95% CI, 0.12–0.5) at four years in patients with esophageal stenosis at baseline whereas it was 0.12 (95% CI, 0.04–0.2) at two years and 0.15 (95% CI, 0.05–0.25) at four years among the remaining cases. On the other hand, radiation dose did not correlate with the cumulative incidence of esophageal perforation (p = 0.85; S1 Fig).

**Table 2:** Univariate analysis of clinical variables associated with esophageal fistula.

Variable	HR (95% CI)	P value
Age (≤ 57 vs. > 57)	1.595 (0.636–4.007)	0.321
Gender (female vs. male)	0.046 (0.000–535.760)	0.520
Body mass index (kg/m^2^) (≤ 21.1 vs. > 21.1)	0.757 (0.312–1.837)	0.538
Body surface area (m^2^) (≤1.61 vs. > 1.61)	1.023 (0.421–2.486)	0.961
ECOG^a^ performance status (0–1 vs. 2–3)	1.165 (0.269–5.047)	0.838
Stage (I&II vs. III)	1.810 (0.528–6.206)	0.345
T stage (1–3 vs. 4)	0.342 (0.135–0.867)	0.024
N stage (0–2 vs. 3)	1.252 (0.516–3.038)	0.619
Tumor location (U^b^ only vs. others)	0.613 (0.242–1.549)	0.300
Esophageal stenosis (no vs. yes)	0.415 (0.171–1.008)	0.052
Total circumferential tumor (no vs. yes)	0.796 (0.183–3.453)	0.760
Tumor length (cm) (≤ 5 vs. >5)	0.825 (0.340–1.998)	0.669
Smoking (no vs. yes)	0.831 (0.192–3.591)	0.804
Alcohol (no vs. yes)	0.452 (0.060–3.384)	0.439
Hypertension (no vs. yes)	1.221 (0.358–4.168)	0.750
Diabetes (no vs. yes)	0.799 (0.232–2.744)	0.721
Chemotherapy regimen (F^c^ vs. NF^d^)	0.472 (0.108–2.056)	0.317
Radiation dose (Gy) (≤ 61.2 vs. > 61.2)	1.092 (0.444–2.685)	0.847

^a^ECOG: Eastern Cooperative Oncology Group.

^b^U: upper thoracic.

^c^F: fluoropyrimidine-based.

^d^NF: not fluoropyrimidine-based.

**Table 3:** Multivariate analysis of clinical variables associated with esophageal fistula.

Variable	HR (95% CI)	P value
Age (≤ 57 vs. > 57)	1.306 (0.509–3.352)	0.579
T stage (4 vs. 1–3)	3.776 (1.383–10.308)	0.010
Tumor location (U^a^ only vs. others)	0.393 (0.146–1.059)	0.065
Esophageal stenosis (yes vs. no)	2.601 (1.053–6.428)	0.038
Chemotherapy regimen (F^b^ vs. NF^c^)	0.419 (0.093–1.894)	0.259

^a^U: upper thoracic.

^b^F: fluoropyrimidine-based.

^c^NF: not fluoropyrimidine-based.

### Clinical course of esophageal fistula

**Fig 3:**
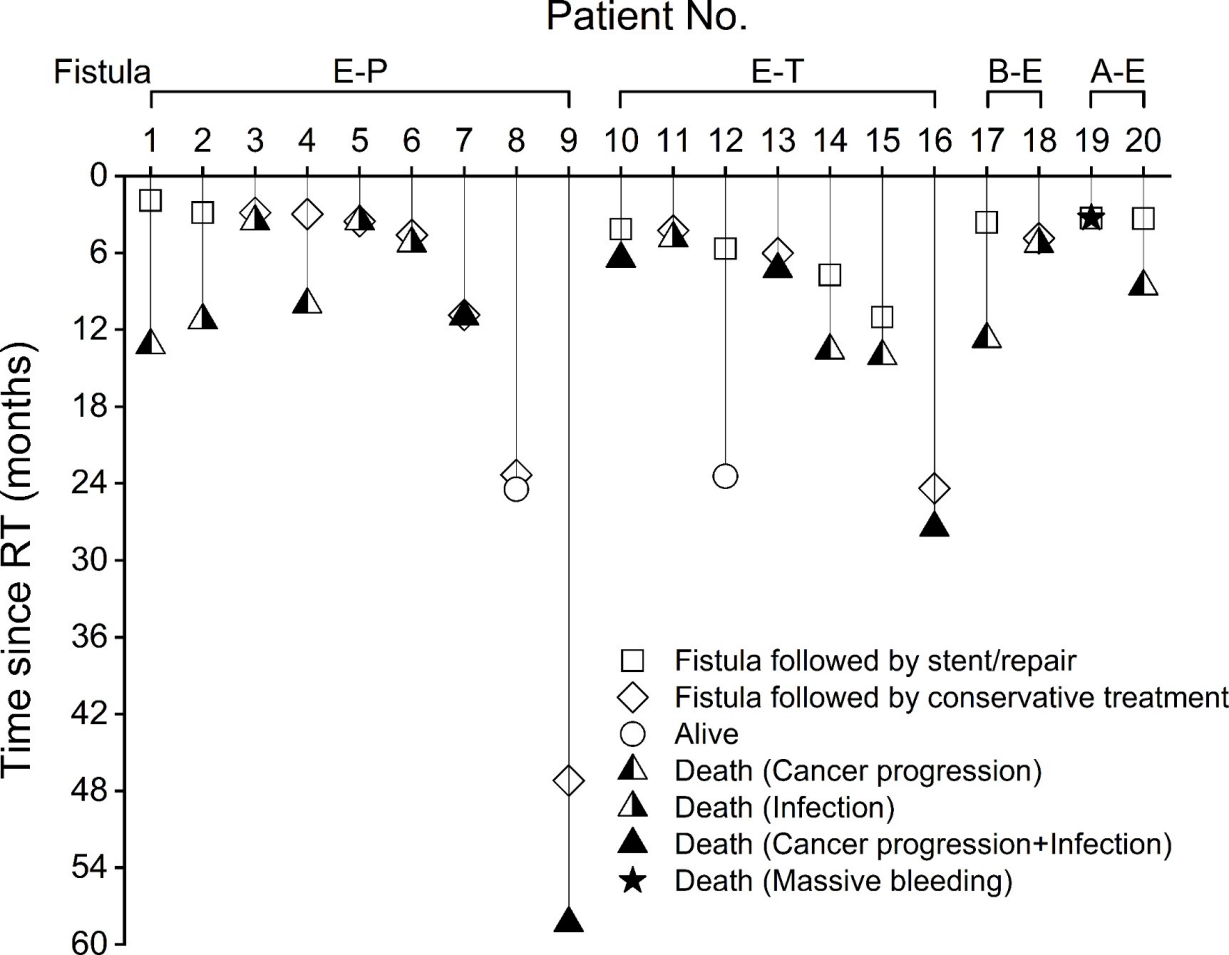
Clinical course of esophageal fistula in 20 cases. A-E, aorto-esophageal. B-E, bronchio-esophageal. E-P, esophago-pleural. E-T, esophago-tracheal.

Fig 3 showed the clinical course of esophageal fistula in 20 patients. The management of the esophageal fistula were mostly proposed by our institutional multidisciplinary esophageal cancer team as the fistula location, perforation severity, cancer control status, medical condition, and other patient personal factors were taken in account. In general, stent placement or surgical repair for the fistula was suggested if possible. In the present study, six patients underwent the airway or esophageal stenting by endoscope with or without fluoroscopy (S2 Table), and three patients received surgical repair for the fistula. On the other hand, the remaining 11 patients had antibiotics and conservative treatments due to the reasons which were listed in S3 Table. Eighteen patients with esophageal fistula died at data cutoff date. The causes of deaths included fistula-related massive hemorrhage in one, cancer progression in six, cancer progression plus fistula-related infection in five, and fistula-related infection in six patients. Among the 20 patients with esophageal fistula, the median survival after the fistula was 3.1 months. There was a trend toward better survival after esophageal fistula among patients receiving repair or stenting for the fistula than those only undergoing conservative treatments (median survival, 5.9 vs. 0.9 months, p = 0.058; S2 Fig).

## Discussion

The present study analyzed 129 ESCC patients undergoing definitive CCRT with IMRT technique. Twenty patients experienced esophageal fistula and had inferior OS. We identified T4 and esophageal stenosis at baseline as independent risk factors of esophageal fistula by multivariate analysis. After the esophageal fistula, there was a trend toward improved survival among patients receiving repair or stenting for the fistula when compared to those only undergoing conservative treatments.

Prior studies have reported esophageal fistula after radiotherapy alone or chemoradiotherapy for esophageal cancer. Notably, several key factors differentiated our data from the previously published ones. To begin with, IMRT was utilized in the present study while conventional technique or 3DCRT were used in the earlier researches [5–9]. In addition, all patients underwent definitive CCRT in the current cohort whereas the previous reports included cases treated with radiotherapy alone [6, 14, 15], sequential chemoradiotherapy [10], brachytherapy [16, 17], or salvage radiotherapy [15, 18–20]. Furthermore, the current cohort only included patients with ESCC, but patients with ESCC and esophageal adenocarcinoma were combined for analysis in some earlier studies [14, 17, 21]. To the best of authors’ knowledge, we were the first to investigate esophageal fistula specifically in ESCC patients treated by definitive concurrent chemotherapy and IMRT. Finally, the clinical course of esophageal fistula in individual patients was reported in the present study and could be more clearly viewed with the time scale.

In the current study utilizing IMRT, 15.5% of our patients experienced esophageal fistula after CCRT for ESCC. This proportion was comparable to 5.3–24.1% in the literature using conventional technique or 3DCRT [5–9]. We further observed that patients with esophageal fistula had inferior OS. In line with the literature [6, 10], we found that T4 acted as a risk factor of esophageal fistula after CCRT. Eleven of the 20 esophageal fistulas communicated with the large airways or aorta in the current cohort. These fistulas possibly developed upon tumor regression after CCRT, and thus treatments of less intensity might be pondered for cases with T4 disease. Accordingly, our data supported the recommendations of National Comprehensive Cancer Network Guidelines which suggest to consider chemotherapy alone for esophageal cancer in the setting of cancer invasion of trachea or great vessels [22]. Furthermore, nine patients had esophago-pleural fistula in the present study. This result reminds to take the risk of pleural perforation into consideration when the treatment plans for ESCC are made. Moreover, consistent with the earlier researches [5, 10], esophageal stenosis independently increased the risk of esophageal fistula in the present study. But, the other studies did not observe the similar finding [6, 7, 20]. These discrepant results possibly derived from various definitions of esophageal stenosis used in the published literature and a limited sample size of each study. In addition, the mechanism by which esophageal stenosis leads to perforation of the esophageal wall is still largely unknown [5, 10]. We speculated that there might be more traumatic injury to the esophageal wall during food swallowing in patients with esophageal stenosis. As a result, esophageal fistula more frequently developed in cases with esophageal stenosis. Overall, the impact of esophageal stenosis on esophageal fistula remains to be further elucidated.

The median survival after esophageal fistula of the current cohort was 3.1 months which was akin to 3.2 months in the previous study [10]. Eleven of 20 patients with esophageal fistula died of cancer progression with or without fistula-related infection in the present study. Our results pointed out the dilemma of restricted salvage treatments for the persistent or progressive tumors in face of CCRT-related adverse events. Notably, we observed a trend toward improved survival after the fistula among patients undergoing repair or stenting for the esophageal perforation when compared to those only receiving conservative treatments. These results suggested the potential benefit of repair or stenting for the esophageal fistula. However, with the limited cases of esophageal fistula in the present study, we could not attribute the trend of survival difference to the management for esophageal perforation or other clinical factors. On the other hand, in a previous prospective study evaluating self-expanding metal stents in the palliation of malignant esophageal obstruction [23], patients who have received radiation therapy may be at greater risk for complications. The adverse events after stent placement should also be taken into consideration when esophageal fistula was treated with stenting. Further investigation is warranted to determine the optimal management for esophageal fistula after CCRT in esophageal cancer.

The current study was limited by the retrospective research design and inherent biases. In addition, the present study only included ESCC patients undergoing definitive CCRT with IMRT technique. The results could not be generalized to patients diagnosed with adenocarcinoma, managed with neoadjuvant CCRT, or treated via radiation techniques other than IMRT. But on the positive side, we provided a specific information regarding esophageal fistula after definitive concurrent chemotherapy and IMRT for ESCC. Further validation with independent cohorts is warranted.

## Conclusions

T4 and esophageal stenosis at baseline independently increased the risk of esophageal fistula in ESCC treated by definitive CCRT with IMRT technique. There existed a trend toward improved survival after the fistula among patients receiving repair or stenting for esophageal perforation.

## Supporting information

S1 Fig Cumulative incidence of esophageal fistula by RT dose. (PDF)

S2 Fig Survival after esophageal fistula by management of the fistula. (PDF)

S1 Table Summary of the chemotherapy regimens and radiation doses. (PDF)

S2 Table Information of stents used to cover esophageal fistula in six patients. (PDF)

S3 Table Treatments and outcomes of esophageal fistula in 20 patients. (PDF)
