# Supplementary figures and images for: Esophageal fistula after definitive concurrent chemotherapy and intensity modulated radiotherapy for esophageal squamous cell carcinoma
Source: PLoS One. 2021 May 14;16(5):e0251811. doi: 10.1371/journal.pone.0251811 (PMC8121322; doi:10.1371/journal.pone.0251811)

**S1 Fig. Cumulative incidence of esophageal fistula by RT dose**

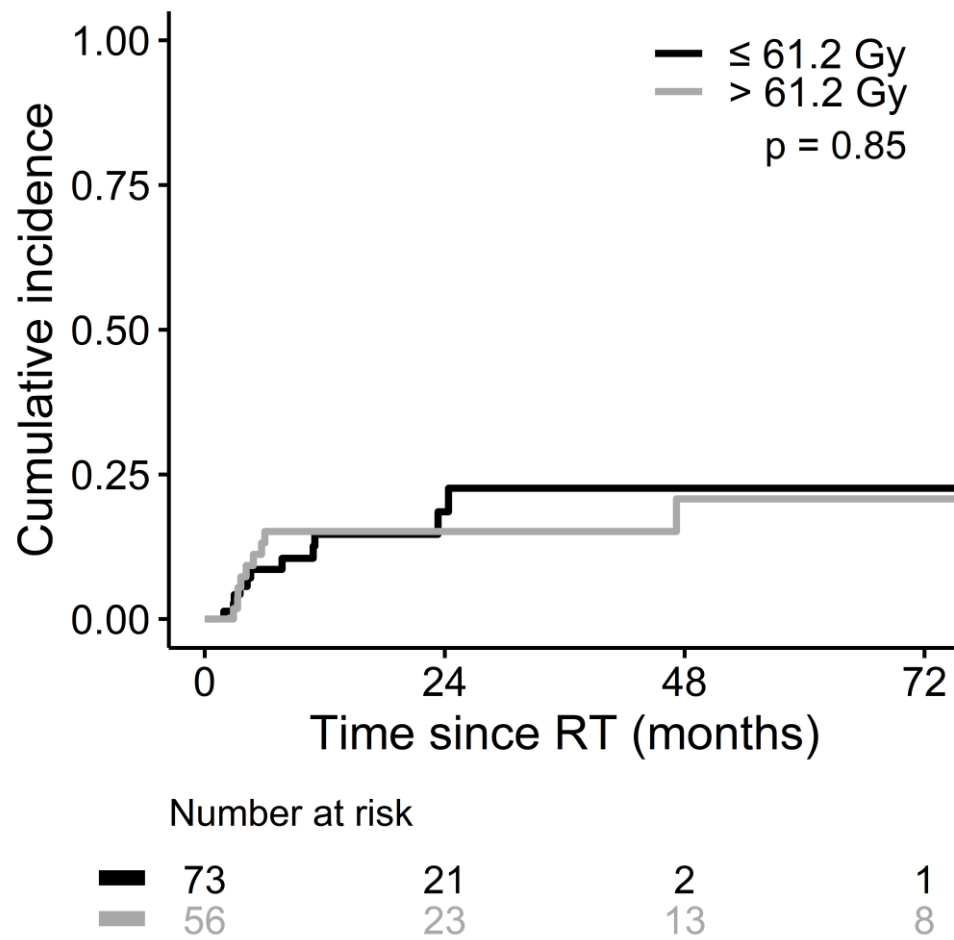

Supplement: S1 Fig — (PDF) [file pone.0251811.s001.pdf]

**S2 Fig. Survival after esophageal fistula by management of the fistula**

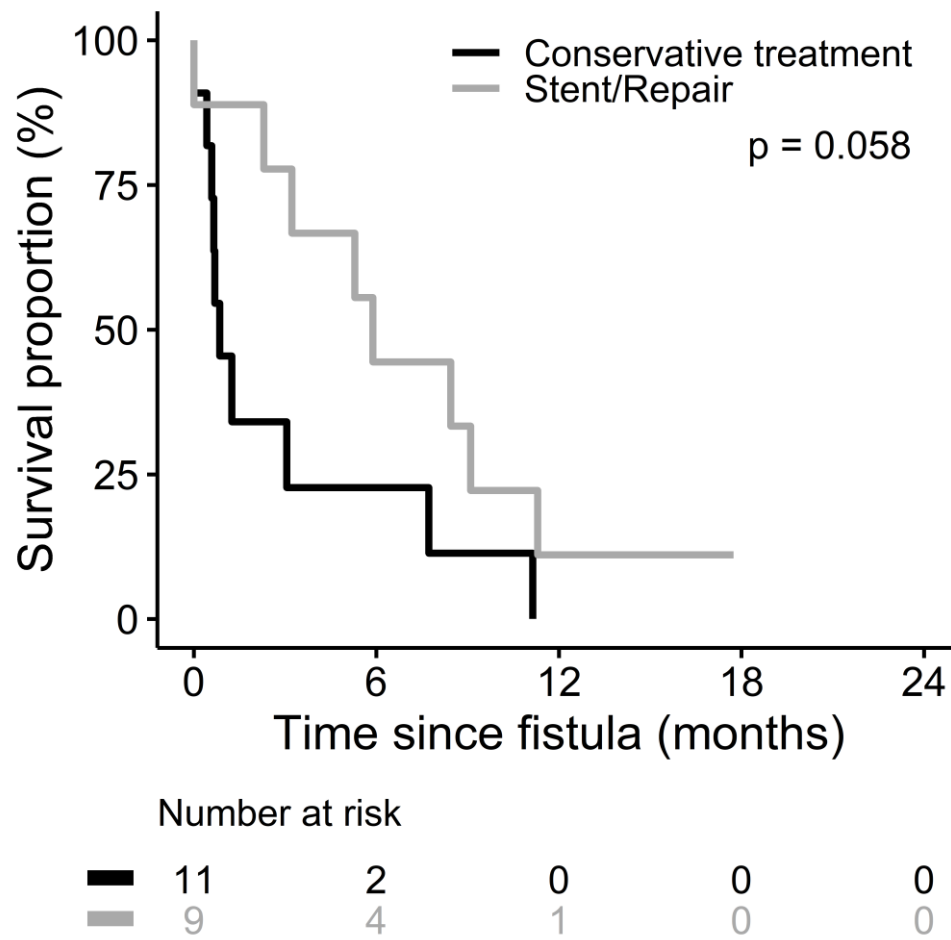

Supplement: S2 Fig — (PDF) [file pone.0251811.s002.pdf]
