# Supplementary material for: Esophageal fistula after definitive concurrent chemotherapy and intensity modulated radiotherapy for esophageal squamous cell carcinoma
Source: PLoS One. 2021 May 14;16(5):e0251811. doi: 10.1371/journal.pone.0251811 (PMC8121322; doi:10.1371/journal.pone.0251811)
Supplement: S1 Table — (PDF) [file pone.0251811.s003.pdf]

S1 Table. Summary of the chemotherapy regimens and radiation doses

Chemotherapy regimen and radiation dose	Number (%) (n = 129)
**Cases with esophageal fistula (n = 20)**	
**Chemotherapy regimen**	
P[a] (25 mg/m^2) + F[b] (1000 mg/m^2) QW[c]	9 (7.0)
P (20 mg/m^2 daily, D[d]1–4) + F (800 mg/m^2 daily, D1–4) Q4W[e]	6 (4.7)
P (20-25 mg/m^2) + F (1500-2000 mg/m^2) + L[f] (150-200 mg/m^2) QW	2 (1.6)
T[g] (80 mg/m^2) + P (25 mg/m^2) QW	1 (0.8)
Docetaxel (35 mg/m^2) + P (35 mg/m^2) + F (1500 mg/m^2) Q2W[h]	1 (0.8)
Docetaxel (35 mg/m^2)	1 (0.8)
**Radiation Dose (Gy)**	
Median (IQR)	61.2 (54-66.6)
**Cases without esophageal fistula (n = 109)**	
**Chemotherapy regimen**	
P (25 mg/m^2) + F (1000 mg/m^2) QW	53 (41.1)
P (20 mg/m^2 daily, D1–4) + F (800 mg/m^2 daily, D1–4) Q4W	24 (18.6)
P (25-40 mg/m^2) + F (2000-2600 mg/m^2) + L (200-300 mg/m^2) Q2W	12 (9.3)
P (20-25 mg/m^2) + F (1500-2000 mg/m^2) + L (150-200 mg/m^2) QW	9 (7.0)
P (25 mg/m^2) + F (2000 mg/m^2) + L (200 mg/m^2) + Cet[i] (400 → 250 mg/m^2) QW	1 (0.8)
P (20 mg/m^2) + F (1600 mg/m^2) + L (160 mg/m^2) + E[j] (30 mg/m^2) QW	1 (0.8)
F (1600 mg/m^2) QW	1 (0.8)
F (2000 mg/m^2) + Carboplatin (AUC 4) Q2W	1 (0.8)
F (1600 mg/m^2) + L (160 mg/m^2) QW	1 (0.8)
T[j] (50 mg/m^2) + Carboplatin (AUC 2) QW	1 (0.8)
T (35 mg/m^2) + P (15 mg/m^2) + Cet (400 → 250 mg/m^2) QW	1 (0.8)
Docetaxel (40 mg/m^2) + P (40 mg/m^2) QW	1 (0.8)
Docetaxel (35 mg/m^2) + Carboplatin (AUC 3) QW	1 (0.8)
P (40 mg/m^2) QW	2 (1.6)
**Radiation Dose (Gy)**	
Median (IQR)	61.2 (54-66.6)

[a]P: cisplatin

[b]F: fluorouracil

[c]QW: weekly

[d]D: day

[e]Q4W: every 4 weeks

[f]L: leucovorin

[g]T: paclitaxel

[h]Q2W: every 2 weeks

[i]Cet: cetuximab

[j]E: etoposide
