# Supplementary material for: Esophageal fistula after definitive concurrent chemotherapy and intensity modulated radiotherapy for esophageal squamous cell carcinoma
Source: PLoS One. 2021 May 14;16(5):e0251811. doi: 10.1371/journal.pone.0251811 (PMC8121322; doi:10.1371/journal.pone.0251811)
Supplement: S2 Table — (PDF) [file pone.0251811.s004.pdf]

S2 Table. Information of stents used to cover esophageal fistula in six patients

**Patient No.**[a]	**Brand**	**Type**	**Diameter x Length**
1	Endo-FLEX Esophageal stent	Partially covered	20 mm x 80 mm
2	Endo-FLEX Esophageal stent	Partially covered	20 mm x 140 mm
10	Ultraflex™ Tracheobronchial stent	Partially covered	14 mm x 40 mm
14	Ultraflex™ Tracheobronchial stent	Partially covered	20 mm x 60 mm
15	Ultraflex™ Esophageal stent	Partially covered	23 mm x 150 mm
17	Ultraflex™ Esophageal stent	Partially covered	23 mm x 100 mm

[a] Patient No. corresponding to those in figure 3.
