# Supplementary material for: Esophageal fistula after definitive concurrent chemotherapy and intensity modulated radiotherapy for esophageal squamous cell carcinoma
Source: PLoS One. 2021 May 14;16(5):e0251811. doi: 10.1371/journal.pone.0251811 (PMC8121322; doi:10.1371/journal.pone.0251811)
Supplement: S3 Table — (PDF) [file pone.0251811.s005.pdf]

S3 Table. Treatments and outcomes of esophageal fistula in 20 patients

Patient No.[a]	Age	Treatment	Reasons for conservative treatment	Survival after fistula (months)
1	56	Esophageal stent		11.30
2	51	Esophageal stent		8.38
3	57	Conservative treatment	Sepsis	0.69
4	56	Conservative treatment	Infection + fistula location	7.06
5	58	Conservative treatment	Infection	0.00
6	48	Conservative treatment	Infection + fistula location	0.66
7	69	Conservative treatment	Infection + terminal stage	0.13
8	70	Conservative treatment	Patient preference	1.12
9	59	Conservative treatment	Economic factor	11.14
10	36	Tracheal stent		2.30
11	64	Conservative treatment	Fistula location + economic factor	0.59
12	61	Surgical repair		17.74
13	57	Conservative treatment	Fistula location	1.25
14	53	Tracheal stent		5.88
15	49	Esophageal stent		3.06
16	62	Conservative treatment	Fistula location	3.06
17	54	Esophageal stent		9.10
18	81	Conservative treatment	Fistula location	0.43
19	57	Surgical repair		0.00
20	47	Surgical repair		5.29

[a] Patient No. corresponding to those in figure 3.
